# Variation in groundwater manganese in Finland

**DOI:** 10.1007/s10653-020-00643-x

**Published:** 2020-07-03

**Authors:** Anne Kousa, Hannu Komulainen, Tarja Hatakka, Birgitta Backman, Sirpa Hartikainen

**Affiliations:** 1 Environmental Solutions, Geological Survey of Finland, Kuopio, Finland; 2 Environmental Solutions, Geological Survey of Finland, Espoo, Finland; 3 Health Security, Environmental Health, National Institute for Health and Welfare, Kuopio, Finland; 4 Faculty of Health Sciences, School of Pharmacy, University of Eastern Finland, Kuopio, Finland

**Keywords:** Manganese, Groundwater, Bedrock, Quaternary deposit, Medical geology

## Abstract

Increasing evidence has emerged that Mn derived from drinking water could be a health risk, especially for children. This study aimed to provide more information on the variation in Mn concentrations in well water and factors that affect manganese concentrations in groundwater in the natural environment. The geochemical data consisted of analyses of single water samples (*n* = 5311) that were taken only once and data from monitoring sites where water samples (*n* = 4607) were repeatedly taken and analyzed annually from the same wells. In addition, the well-specific results from six wells at monitoring sites were described in detail. We obtained the data on water samples from the groundwater database of Geological Survey of Finland. In single samples, Mn concentrations varied from < 0.02 µg/l to 5800 µg/l in bedrock well waters and up to 6560 µg/l in Quaternary deposit well waters. Results from single water samples from bedrock wells and Quaternary deposit wells indicated that the dissolved oxygen content has an inverse association with the Mn concentration. When the dissolved oxygen O_2_ levels were lower, the Mn concentrations were higher. No clear association was found between the Mn concentration and the pH or depth of the well for single samples. Part of Mn was particle bound, because total Mn was higher than soluble Mn in most measured samples. In the monitoring survey, large variation in Mn concentrations was found in bedrock well water in Kemijärvi, 114–352 µg/l, and in dug well water in Hämeenkoski, 8.77–2640 µg/l. Seasonal and spatial variability in Mn concentrations in water samples from two bedrock wells was large at monitoring sites in northern Finland. Variability in the Mn concentrations in groundwater can be large, even in the same area. These data suggest that single measurements of the Mn concentration from a water source may not reveal the Mn status, and measurement of both the total and soluble Mn concentrations may be recommended.

## Introduction

Manganese (Mn) is a naturally occurring element found ubiquitously in bedrock, soil, water, air, and plants. Manganese may also be present in the environment as a result of anthropogenic sources such as mining, industrial emissions, and landfill leaching. In groundwater and surface water sources, manganese typically occurs particularly in anaerobic conditions (WHO 2017). The main oxidation states in water are Mn(II), which is a soluble and bioavailable form, and Mn(IV), which is an insoluble form. Mn(II) usually predominates in waters at a pH level of 4–7. If the pH and redox potential of the water increase, Mn(II) may be oxidized to Mn(IV). The main factors contributing to the chemical composition of groundwater are geological, atmospheric, marine, anthropogenic, and seasonal factors (Lahermo et al. 1990). Geological factors contribute to the composition of groundwater through the effect of chemical water–rock interactions in aquifers (Lahermo et al. 1990). The groundwater in deep bedrock aquifers has a longer residence time and displays smaller seasonal variations than shallow aquifers (Kortelainen and Karhu 2004). Private wells have usually been dug into glacial till (soil parent material) deposits or drilled into fractured bedrock in sparsely inhabited rural areas in Finland (Lahermo et al. 1990; Korkka-Niemi 2001). High Mn concentrations in groundwater are especially found in the clay-rich western coastal areas of Finland, where acid sulfate areas prevail (Tarvainen et al. 2001). At levels exceeding 100 µg/l, Mn in water supplies may cause an undesirable taste in drinking water and stain sanitary ware and laundry (WHO 2017). The presence of Mn in tap water, similarly to iron, may lead to the accumulation of deposits in the distribution system. In household water, concentrations below 100 µg/l are usually acceptable to consumers. At a level of 200 µg/l, manganese will often form a coating on pipes, which may slough off as a black precipitate (WHO 2017). In Finland, household water is derived from groundwater and surficial water. About 60% of water is distributed by municipal waterworks, which is derived from groundwater or artificial groundwater. Mn is not a major problem in public water supplies.

Mn is known as a neurotoxic element, but it is also an essential micronutrient required in trace amounts for human health. The general population (i.e., having no occupational exposure) may become exposed to manganese through the ingestion of food and water, the inhalation of dust, and to a lesser degree through dermal contact with air, water, and soil (ATSDR 2012). The main dietary sources of Mn are tea, nuts, whole grains, legumes, and fruits (ATSDR 2012).

Manganese concentrations in groundwater are dependent on rainfall chemistry, the dissolution of minerals from bedrock, leaching by water percolating through soil, and the residence time. Higher concentrations of Mn are found in acidic groundwater with a low pH and anaerobic conditions (Lahermo et al. 1990; IMnI 2013). Aquifer properties, such as the occurrence of peat, fine-grained sediments, or clay, the condition of the well, and anthropogenic pollution, are more pronounced contributors to the Mn concentrations in groundwater than the lithological environment (Lahermo et al. 1990).

To date, Mn in water has only been considered as a technical/aesthetic or cosmetic problem at the regulatory level. However, increasing evidence has emerged that Mn derived from drinking water could be a health risk, especially for children (Bouchard et al. 2011; Khan et al. 2011; Wasserman et al. 2011; Neal and Guilarte 2013; Oulhote et al. 2014). The risk of neurotoxic effects on children starts to increase when the concentration in drinking water exceeds 100 μg/l (Bouchard et al. 2011). Higher Mn levels in tap water and hair were significantly associated with lower intelligence quotient (IQ) scores among school children at the age of 6–13 years in a Canadian study (Bouchard et al. 2011). The median Mn concentration in their tap water was 34 μg/l (range 1–2700 µg/l). A tenfold increase in drinking water Mn concentrations was associated with a decrease of 2.4 IQ points (95% confidence interval: − 3.9 to − 0.9; *p* < 0.01) (Bouchard et al. 2011). This association was not found with Mn intake from food, suggesting that exposure from drinking water and food may differ (Bouchard et al. 2011). Manganese may more readily be absorbed from drinking water than when ingested via food (Health Canada 2019). Even at low levels, exposure to manganese in tap water was associated with poorer neurobehavioral performances in children (Oulhote et al. 2014). Smith et al. (2013) demonstrated a strong relationship between blood manganese and iron deficiency among children. Children with iron deficiency anemia may be at risk of manganese toxicity (Smith et al. 2013).

Guidelines for Mn in drinking/household water vary between countries. In Finland, there is no health-based chemical quality standard requirement for Mn in household water. The quality recommendation, based on technical/aesthetic effects, is below 50 µg/l, and the threshold limit is 100 µg/l for small units or households using private wells (STM 1352/2015, STM 401/2001). The health-based limit value for Mn set by the World Health Organization is 400 µg/l, based on an upper tolerable intake (WHO 2011). According to WHO, the calculated health-based value is well above concentrations of Mn normally found in drinking water. Therefore, it was not considered necessary to derive a formal guideline value in the most recent evaluation (WHO 2011).

A good understanding of the natural variability in the concentrations of inorganic elements, including Mn, in private water supplies is important to ensure safe potable water for those residents whose water source is solely a private well (Ander et al. 2016). To the best of our knowledge, studies on factors associated with the natural concentrations of Mn in groundwater from the human health risk perspective are scarce. Therefore, a more detailed evaluation of the spatial and temporal variation in Mn concentrations in groundwater is needed for reliable intake and health risk assessment. The purpose of this paper is to provide further information on the factors that affect manganese concentrations in groundwater in the natural environment. This article is mainly based on data from a project published in Archive report 95/2016 of the Geological Survey of Finland by Kousa et al. (2017, in Finnish).

## Material and methods

The Precambrian bedrock of Finland is composed of igneous and metamorphic rocks (Bedrock of Finland DigiKP, 2019). The bedrock is overlain by Quaternary deposits, a glacial overburden with an average thickness of about seven meters. Quaternary deposits were formed by several glaciations during the last two to three million years. These surficial loose materials are composed of sand, gravel, clay, and silts, which are extensively covered by recent peat layers (Koljonen and Tanskanen 1992). The studied dataset contains analyses of groundwater samples. However, water samples were taken from different kinds of wells. Water samples of springs, captured springs, and dug wells are often taken directly from the well or the waterbed. Thus, in these water samples, the structure of the captured spring or well might have an effect on the water quality. Water samples from bedrock wells are usually taken from a tap (drinking water/tap water). Therefore, the groundwater quality of these samples may be affected by the water system. In these cases, water flows through water pipes as well as a pressure tank, and these might affect the metal concentrations in the water. A detailed examination of the bedrock geology was not conducted for the wells in the present study.

In rural areas, water supplies consist of wells drilled into bedrock, dug wells, springs, captured springs, and sometimes groundwater tubes and wells drilled into overburden discharging household water from Quaternary deposits. Dug wells, which are often located in glacial till areas, represent the most common well type in rural areas (Backman 2004; Korkka-Niemi 2001). Formerly, dug wells were lined with wood or stones, but nowadays they are mainly constructed of concrete rings (Korkka-Niemi 2001). Springs and captured springs are natural sources of water flowing from an aquifer to the earth’s surface. Captured springs are lined with a shallow wooden casing or concrete ring (Korkka-Niemi 2001). Drilled wells obtain water from either overburden or bedrock aquifers. Wells drilled into overburden are constructed into overburden aquifers, i.e., geological materials above bedrock (Simpson 2015). Quaternary deposit wells are usually shallow, only 3–5 m in depth, and 80–120 cm in diameter. Springs and shallow dug wells a few meters in depth represent shallow groundwater percolating through the ground surface (Tarvainen et al. 2001). Likewise, wells drilled into fractured bedrock are common. These wells are generally 40–80 m deep and 110 mm in diameter. Their average yield is 500–2000 L per hour (Korkka-Niemi 2001; Lahermo et al. 1990). Groundwater observation tubes, usually made of PVC or PEH plastic, are drilled into overburden to monitor groundwater quality.

This study aimed to clarify the variability in Mn concentrations and the relationship with other geochemical factors of groundwater and properties of water sources in Finland, utilizing data collected by GTK during 1992–2013. Two datasets of water sample analyses were available: single samples taken once from wells and samples taken repeatedly from the same wells at monitoring sites. Data on bedrock wells and wells in Quaternary deposits (dug wells, springs, captured springs, and tube wells) were included in this study. All these water samples had been analyzed using ICP-MS/AES with a detection limit of 0.02 µg/l for Mn. The groundwater data used in this study consisted of water samples analyzed after 1991, because the analysis methods changed and ICP-MS/AES was solely in use after that. Analytical data before 1992 were not comparable with ICP-MS/AES analyses, because the detection limits differed. The pH values and the concentrations of dissolved oxygen (O_2_) were measured in the field in conjunction with water sampling by using oxygen and pH meters (e.g., WTW Oxi 320/330 and WTW Multiline P3 pH/LF, respectively). The samples were taken by experienced and/or certified personnel. The quality of sampling was assured by including blank samples and/or duplicate samples in the analysis. Water samples were analyzed in accredited laboratories, and their analytical methods were accredited or were based on standards. The accredited testing laboratories enforce their own customary quality assurance methods. The Kolmogorov–Smirnov test was used to verify the normality of the distribution of groups classified according to Mn concentrations. Statistical differences between the groups were tested using the nonparametric Kruskal–Wallis test and ANOVA, and correlations between variables were determined by two-tailed Spearman correlation. Statistical analyses were conducted, and graphics were produced using IBM® SPSS® Statistics versions 24 and 25 and map presentations using ArcMap 10.3 software.

*Single water samples* were taken only once from each well during 1992–2013. We obtained data on single water samples from the groundwater database of the Geological Survey of Finland (GTK). The total number of single water samples was 5311, including samples from 2383 bedrock wells and 2928 Quaternary deposit wells in Finland. The data were classified into four groups based on the Mn concentrations according to the Finnish national quality recommendations and the guidelines of WHO for statistical analyses (STM 1352/2015, STM 401/2001, WHO 2011): < 50 µg/l, 51–100 µg/l, 101–400 µg/l, and > 400 µg/l.

**Fig. 1:**
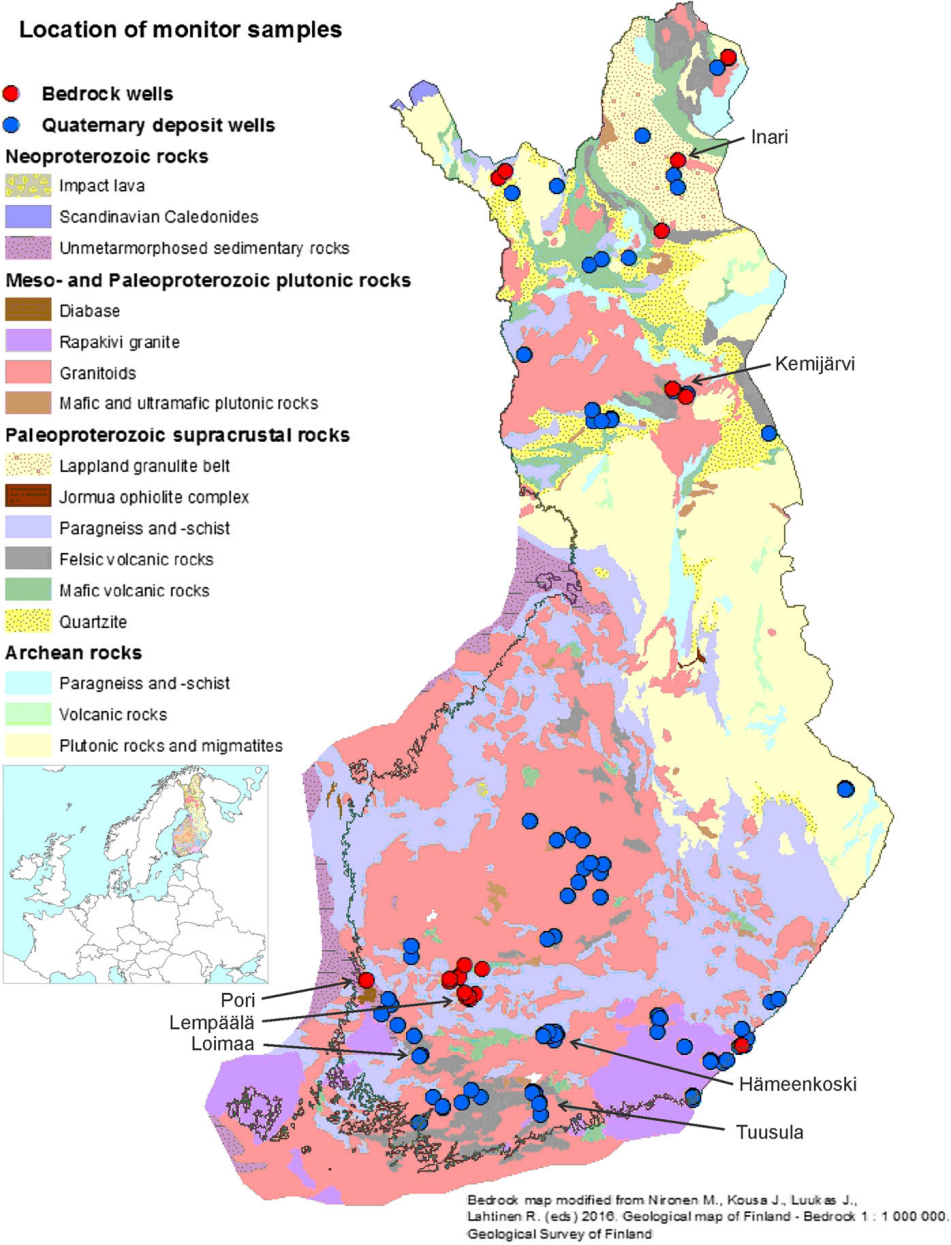
Locations of sampled monitoring sites and index map

*Water samples from monitoring sites* were repeatedly taken and analyzed annually from the same wells during 1992–2009. We obtained data on monitoring sites from the groundwater database of the Geological Survey of Finland (GTK). GTK has conducted groundwater monitoring in southern Finland since 1969 (Backman 1993). The monitoring wells are mostly located in southern Finland and to a lesser extent in the other parts of the country (Fig. 1). Since 1993, monitoring sites have expanded to cover virtually the whole country (Backman et al. 1999; Backman 2004). The acid soil areas from the western coast of Finland have not been included in the monitoring sites of GTK. The latest water samples included in this study were taken in 2009. The original aim of the groundwater monitoring survey conducted by GTK was to investigate the short- and long-term effects of geology and human activities on groundwater quality. In this study, the total number of water samples was 4607 from 138 wells at monitoring sites, consisting of 380 water samples from bedrock wells (*n* = 22) and 4227 samples from Quaternary deposit wells (*n* = 116). Data on the monitoring sites selected in this study covered such a long period that the seasonal and annual variations could be detected. Annual variations in rainfall and temperature have had an effect on groundwater formation and levels and therefore also on the quality of groundwater. The representation of different geological environments and well types was also taken into consideration when monitoring sites were selected.

## Results and discussion

The Finnish national maximum recommendation for Mn in drinking water is < 50 µg/l and for small units and private wells < 100 µg/l (STM 1352/2015, STM 401/2001). In single water samples, about 41% of single water samples from wells drilled in bedrock and 17% of those from wells in Quaternary deposits exceeded the Finnish national recommendation for Mn. Twenty-eight per cent of waters in bedrock wells and 11% in Quaternary deposit wells exceeded the national recommendation for small units and private wells. In monitoring sites, the maximum recommendation for small units was exceeded in 41.5% of bedrock well water samples and 5.1% of Quaternary well water samples. Mn concentrations in water samples from bedrock wells and Quaternary deposit wells have been presented separately (Table 1), because the geochemistry of groundwater varies between these well types. The wells in Quaternary deposits were mainly dug wells, springs, captured springs, and tubes. The median Mn concentration was 2.31 µg/l in water from all wells in Quaternary deposits and 26.8 µg/l in water from bedrock wells. In the results of the “One Thousand Wells” groundwater quality mapping project in Finland in 1999, the median Mn concentration was 4.35 µg/l in well waters from Quaternary deposits and 16.3 µg/l in wells drilled in bedrock (Lahermo et al. 2002).

**Table 1:** Minimum, median, and maximum Mn concentrations (µg/l) in combined data of single water samples and samples from monitoring sites according to the well type in Finland in 1992–2013

Groundwater Mn µg/l	Minimum	Median	Maximum	Number of samples
*Quaternary deposit*				
Springs	< 0.02	0.56	3760	2195
Captured springs	< 0.02	1.59	2530	657
Groundwater tubes	0.08	0.97	429	1242
Tube wells in aquifers covered by clay	< 0.02	2.49	6560	241
Dug wells	0.02	5.93	5330	2771
*Bedrock*				
Drilled wells	< 0.02	26.8	5800	2671

### Bedrock wells: single water samples

**Fig. 2:**
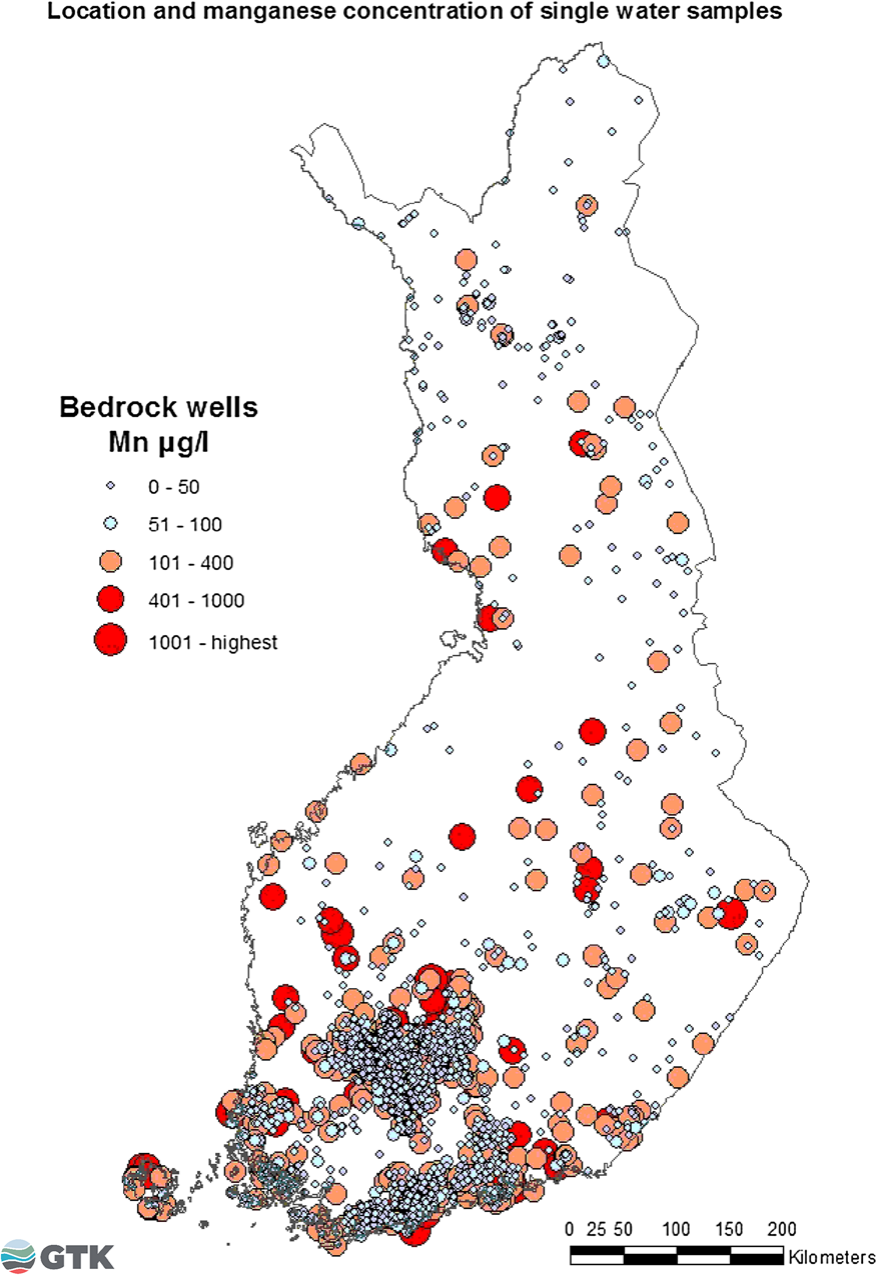
Locations and distribution of Mn concentrations in single water samples from bedrock wells in 1992–2013

A total of 2383 wells drilled into bedrock were included in this study. Anomalously high Mn concentrations in bedrock well water were uniformly distributed in Finland, as shown in Fig. 2. The range of Mn concentrations in waters of wells drilled in bedrock was large, < 0.03–5800 µg/l. Mn correlated significantly with Fe (*r* = 0.624**, *p* < 0.01). The statistics for Mn, iron (Fe), pH, and oxygen (O_2_) content are presented in Table 2.

**Table 2:** Median, mean, standard deviation, maximum, and 2% and 98% percentiles for manganese (Mn), iron (Fe), pH, and oxygen (O_2_) content in single water samples from wells drilled in bedrock

Bedrock drilled wells	Mn µg/l	Fe mg/l	pH (field)	O_2_% (field)
2%	0.25	< 0.02	5.8	8.5
Median	28.4	0.04	7.30	47.6
Mean	100	0.46	7.27	49.1
SD	224	1.75	0.77	26.2
98%	638	5.2	8.84	104
Maximum	5800	39.9	9.50	143
*N*	2291	2291	1331	1134

Statistically significant associations were found between Mn and Fe (*p* = 0.00) and between Mn and O_2_ (*p* = 0.00) when the data were compared for groups classified according to the Mn concentration (Table 3). The Fe concentration increased accordingly with the Mn levels, the median being 0.75 mg/l (750 µg/l) when the Mn concentration was over 400 µg/l. Overall, the median concentration of Fe was 0.04 mg/l (40 µg/l) in this dataset. The Finnish quality recommendation for Fe is below 200 µg/l (STM 2015). The content of dissolved oxygen decreased as a function of increasing Mn concentration up to a Mn concentration 400 µg/l, being 35.7% at lowest. In the highest class of Mn, the pH was lowest, but the difference between the groups was not statistically significant (*p* = 0.69) (Table 3).

**Table 3:** Median, mean, standard deviation (SD), and range of single water samples (n) for Fe, pH, and O_2_ in waters of bedrock wells according to the Mn concentration category

Mn	Fe mg/l^a^	pH (field)	O_2_% (field)^a^
< 50 µg/l			
Median	< 0.03	7.30	56.2
Mean	0.06	7.27	56.0
SD	0.15	0.88	26.3
Range	< 0.03–2.63	5.20–9.50	0.0–144
* n*	1346	792	666
51–100 µg/l			
Median	0.09	7.50	39.2
Mean	0.34	7.36	40.0
SD	0.87	0.60	23.2
Range	< 0.03–7.86	5.20–8.80	0.0–126
* n*	293	166	141
101–400 µg/l			
Median	0.24	7.30	35.7
Mean	1.11	7.26	39.1
SD	2.45	0.56	22.8
Range	< 0.03–28.5	5.40–9.40	6.10–124
* n*	534	297	262
> 400 µg/l			
Median	0.75	7.20	39.2
Mean	2.32	7.16	38.1
SD	4.97	0.46	20.1
Range	< 0.03–39.9	6.20–9.10	9.10–116
* n*	113	71	61

^a^Kruskal–Wallis test, *p* = 0.00

**Fig. 3:**
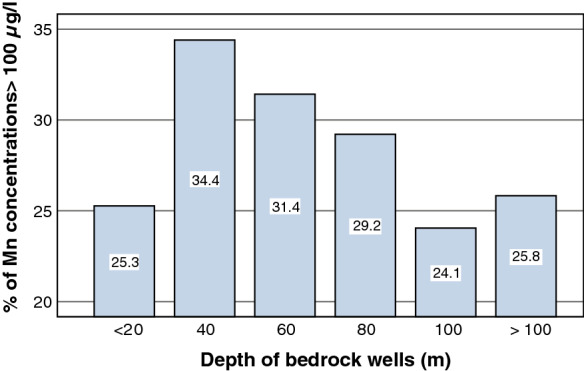
The percentage of single water samples with a Mn concentration above 100 µg/l in each depth class of bedrock wells

The total number of high Mn concentrations, exceeding 100 µg/l, was 592. Within the depth categories of wells, the highest proportion of high concentrations (34.4%) was found in wells with a depth of 20–40 m (Fig. 3). However, the proportion was rather similar (24.1–34.4%) in all depth categories, irrespective of the depth of the bedrock well (*p* = 0.537). Prediction maps of Mn concentrations indicated that the elevated level of Mn in the groundwater of North Carolina in the USA was not attributable to the bedrock geology or well depth (Johnson et al. 2018). Likewise, in our study, high Mn concentrations were found in all depth classes of the bedrock wells studied.

The data indicated that the dissolved oxygen content has an inverse association with Mn concentrations. In the presence of low O_2_ levels, the Mn concentrations were higher. Moreover, Mn was associated with the Fe concentration in water, and high concentrations of both were concomitantly detected. No clear association was found between the Mn concentration and pH in single water samples from bedrock wells.

High Mn concentrations were more often detectable in water from bedrock wells than Quaternary deposit wells. The chemical composition of bedrock water differs from that of shallow groundwater. The retention time and aquifer type have a clear effect on the metal concentrations, including Mn, in groundwater. A high Mn concentration is a result of a long retention time of water passing through geological materials, as well as the reducing conditions prevailing in bedrock wells. This process mobilizes manganese in the soluble Mn(II) form (Korkka-Niemi 2001). Therefore, bedrock groundwater often more contains dissolved elements. In this study, the highest Mn concentrations (> 100 µg/l) in groundwater from drilled bedrock wells were recorded in those wells located in metamorphic schist and paragneiss areas originating from both sedimentary and volcanic sources in the southern coastal area, and in similar geological areas in central and northern Finland. Furthermore, high Mn concentrations were found in rapakivi granite areas in southern Finland. However, the detailed bedrock geology of the wells was not examined in this study.

### Bedrock wells: monitoring survey

Altogether, 380 analyses from 22 bedrock wells in the monitoring survey were included in this study. High Mn concentrations, exceeding 100 µg/l, were found during the monitoring period in four wells. Two of these were located in northern Finland, in the municipalities of Kemijärvi and Inari, which were monitored from 1993 to 2009. The other two wells were located in the southern part of the country: one in the municipality of Lempäälä, monitored from April to August 2005, and the other in the city of Pori, monitored from 1993–2000 (Fig. 1).

**Fig. 4:**
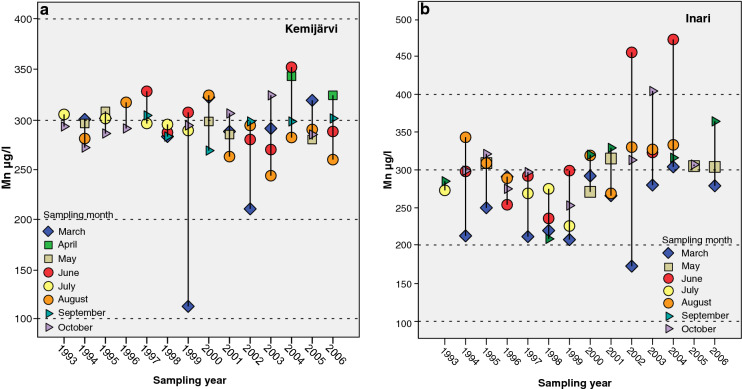
**a and b** Annual and monthly variation in manganese concentrations in water from bedrock wells at the monitoring sites in Kemijärvi (min = 114, max = 352 µg/l) and Inari (min = 173, max = 472 µg/l), northern Finland, in 1993–2006

In Kemijärvi, the bedrock of the study area is composed of Paleoproterozoic granites and variously migmatized granitoid units (Bedrock of Finland DigiKP; Nironen et al. 2016). The sampled well was drilled through a glacial esker. The depth of the well was 83 m, and the thickness of the overlay material was 16 m. The number of water samples was 51. The concentrations of all other elements in the well water except Mn were mainly very low, even below the detection limits. Only the Mn concentration was above the current drinking water regulation of 100 µg/l. The annual variability in Mn concentrations in the well water was between 114 and 352 µg/l in 1993–2006. The variability was lower in the first years of monitoring, from 1993–1998. The largest variation was found in 1999, when the lowest Mn concentration was 114 µg/l in March and the highest was 307 µg/l in June. According to the SiO_2_ concentrations in the same sample, the retention time of the water was clearly shorter than it used to be at this monitoring site. This, as well as the elevated oxygen concentration in the water, may explain the low Mn concentration in 1999. However, the reason for the freshwater pulse in the well is unknown. In general, the variability was greater after 1999 than in earlier years at the Kemijärvi monitoring site (Fig. 4a).

At Inari, the bedrock of the study area is composed of granate–cordierite-rich paragneiss of the Lapland granulite complex (Bedrock of Finland DigiKP; Nironen et al. 2016). The depth of the well was 76 m, and the number of water samples was 51. The lowest Mn concentrations were generally found in samples taken in March (Fig. 4b). The largest variation was between the Mn concentration of 173 µg/l recorded in March 2002 and 472 µg/l in June 2004. The concentrations of some other elements were notably high in this well water. For example, the concentrations of strontium (median 562 µg/l) were tenfold higher than at some other monitoring sites in northern Finland (Hatakka et al. 2007; Backman et al. 1999).

In general, seasonal variation was not evident in the bedrock wells. However, according to the results from these bedrock wells located at monitoring sites, Mn concentrations in two wells displayed clear annual and seasonal variation. High Mn concentrations and considerable annual and monthly variation were found in the waters of wells in both Kemijärvi and Inari in the course of the monitoring. In both wells, Mn concentrations in water samples taken in spring and summer were higher than in samples taken in winter. In Finland, precipitation is usually lower and evaporation is higher in summer, and the amount of water is therefore lower and the level of the groundwater table is low. This could partly explain the variability in Mn concentrations in these two wells.

**Fig. 5:**
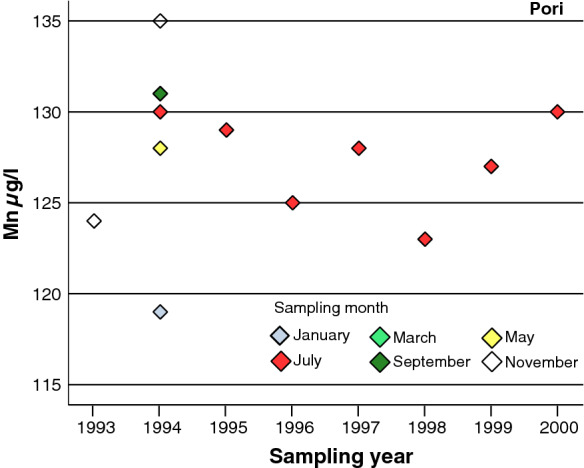
Annual and monthly variation (1994) in the Mn concentration of water from an artesian bedrock well at the Pori monitoring site (min = 119 µg/l, max = 135 µg/l) in southern Finland between 1993 and 2000

The bedrock well in Pori was a 600-m-deep artesian well drilled by GTK. In an artesian well, the water rises to the surface under its own pressure. GTK drilled this well in a Mesoproterozoic sandstone area to intersect the Satakunta sandstone formation and reach the underlying bedrock of the Pirkanmaa migmatite suite (Bedrock of Finland, DigiKP; Nironen et al. 2016). However, the bottom of the sandstone deposit was not reached, and the well was thus entirely situated in sandstone. Annual or monthly variation (in 1994) in Mn concentrations was not very large, being between 119–135 µg/l (Fig. 5).

**Fig. 6:**
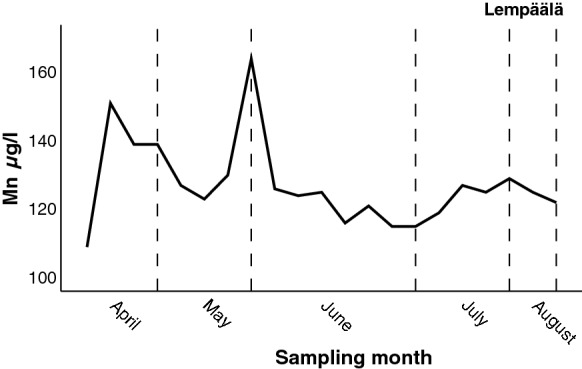
The variation in Mn concentrations in bedrock well water during spring and summer 2005 at the Lempäälä monitoring site (min = 109 µg/l, max = 164 µg/l)

The bedrock well in Lempäälä was located in the unit of the Pirkanmaa migmatite suite. The bedrock of the Lempäälä area mainly consists of mica schist and mica gneiss, with mafic metavolcanic rock interlayers and plutonic intrusions such as granodiorites and granites (Bedrock of Finland, DigiKP; Nironen et al. 2016). The quality of the bedrock well water was monitored from April to August in 2005 by taking 18 water samples and 3 reference samples. The Mn concentration fluctuated between 109 µg/l and 164 µg/l over the five-month period in 2005 (Fig. 6). Detailed information on the pumping volume from wells is not generally available, but in this case, it was possible to roughly estimate the historical water consumption based on information received from the well owners. The well water was used by a market garden. The variability in the Mn concentration of the water was quite low, despite heavy pumping. At the beginning of the test in April, the pumped water volume was low, being 4.1 m^3^/month. Later, the pumped water volume increased (May: 5.6 m^3^/month; June: 263 m^3^/month; July: 228 m^3^/month; and August: 199 m^3^/month) (Backman et al. 2006). The depth of the well was unknown, but the composition of the water corresponded to a long retention time, indicating that the well was deep.

**Fig. 7:**
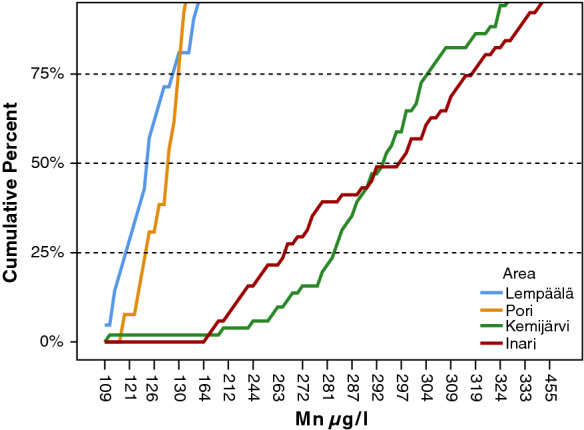
The cumulative percent distribution of manganese concentrations in water for each bedrock well in which the concentration exceeded 100 µg/l at four monitoring sites in southern and northern Finland in 1992–2006

The cumulative frequency distributions of Mn concentrations in waters from four wells drilled into the bedrock in the different parts of Finland are illustrated in Fig. 7. Mn concentrations in bedrock wells at both Lempäälä and Pori in southern Finland were quite stable during the follow-up, as seen in the sharp cumulative curves (Fig. 7). The 50^th^ percentiles of the Mn concentrations at Lempäälä and Pori were 125 µg/l and 128 µg/l, respectively. The flat cumulative curves illustrate that the Mn concentrations were higher and the range larger in both wells in northern Finland, at Kemijärvi and Inari. The 50^th^ percentiles of the Mn concentrations were 294 µg/l and 297 µg/l, respectively (Fig. 7). However, the maximum Mn levels in waters of the other bedrock wells in the same municipalities, Kemijärvi and Inari in northern Finland, were under the maximum quality recommendation of 50 µg/l for Mn.

Spatial variability between areas in northern and southern parts of the country may be associated with different atmospheric conditions (e.g., meltwater from snow) and geological factors such as the lithological composition of the aquifer material and residence time of the water. Taken together, these detailed data suggest that Mn levels may vary in well waters, and analysis of the Mn concentration in a single sample from a well may not reveal the true average Mn level in the water.

### Quaternary deposit wells: single water samples

**Fig. 8:**
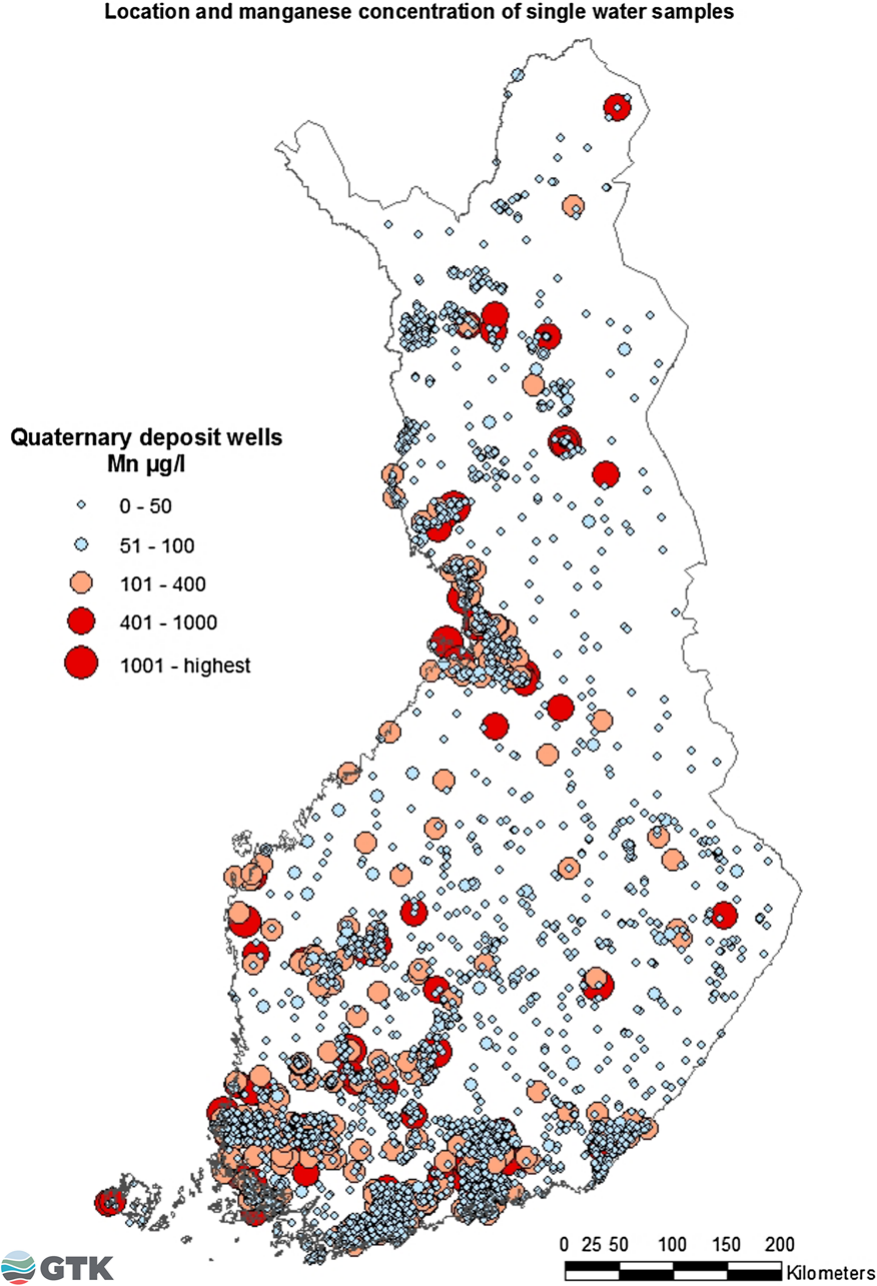
Sampling locations and distribution of Mn concentrations in water samples from Quaternary deposit wells in 1992–2009

Analyses of 2928 single water samples taken after 1992 from wells in Quaternary deposits were included in this study. Anomalous Mn concentrations in groundwater were uniformly distributed across the country (Fig. 8). Statistically significant correlations were found between Mn and Co (*r* = 0.655**, *p* < 0.01) and between Mn and Fe (*r* = 0.566**). Seventeen percent of waters in Quaternary deposit wells exceeded the Finnish national quality recommendation of < 50 g/l for Mn (STM 1352/2015). Eleven percent exceeded 100 µg/l, the maximum recommendation for Mn in small units and private wells (STM 401/2001), and 3% exceeded 400 µg/l, the health-based limit value presented by WHO (2011). The lowest median concentration of Mn, 0.98 µg/l, was detected in springs, while the highest median was in tube wells drilled in Quaternary deposits, being 49.1 µg/l (Table 4).

**Table 4:** Median, mean, standard deviation, maximum, and 2% and 98% percentiles for manganese (Mn), iron (Fe), acidity (pH), and oxygen (O_2_) in single water samples from Quaternary deposit wells

Well type	Mn µg/l^a^	Fe mg/l^a^	pH (field)^b^	O_2_% (field)^b^
Springs				
2%	0,02	< 0.02	5.2	23.0
Median	0.98	0.03	6.60	84.5
Mean	24.9	0.21	6.60	85.4
SD	153	0.99	0.78	29.6
98%	292	1.78	8.3	146
Max	2 920	11.6	9.00	156
* n*	530	530	505	394
Captured springs				
2%	0.05	< 0.02	5.2	8.18
Median	2.70	0.03	6.10	75.4
Mean	31.1	0.12	6.16	76.2
SD	163	0.44	0.52	32.5
98%	388	1.46	7.4	145
Max	2 530	5.27	8.50	156
* n*	318	318	309	281
Dug wells				
2%	0.41	< 0.02	5.4	8.77
Median	8.23	0.04	6.30	64.0
Mean	58.2	0.22	6.36	64.7
SD	223	1.09	0.54	30.1
98%	510	1.63	7.6	131
Max	5 330	31.1	9.00	343
* n*	1931	1930	1686	1533
Tube wells				
2%	< 0.02	< 0.02	5.46	5.37
Median	49,1	0.08	7.00	38.2
Mean	468	5.29	6.96	47.6
SD	1153	20.7	0.59	29.6
98%	5 710	121	8.02	116
Max	6 560	133	8.20	133
* n*	107	107	79	90
Total				
2%	0.07	< 0.02	5.3	7.26
Median	5.83	0.03	6.30	68.5
Mean	64.3	0.39	6.40	69.0
SD	309	4.20	0.62	31.6
98%	553	2.14	7.9	137
Max	6 560	133	9.00	343
* N*	2886	2885	2579	2298

^a^Kruskal–Wallis test, *p* = 0.00

^b^ANOVA, *p* = 0.00

The highest medians of Mn and Fe concentrations were recorded in tube wells drilled in Quaternary deposits (*p* = 0.00). The most acidic well water was found in captured springs (median pH 6.10), while the highest pH, 7.0, was recorded in tube wells (*p* = 0.00). The dissolved oxygen level was highest in springs (median 84.5%) and lowest in tube wells (38.2%) (*p* = 0.00) (Table 4).

**Fig. 9:**
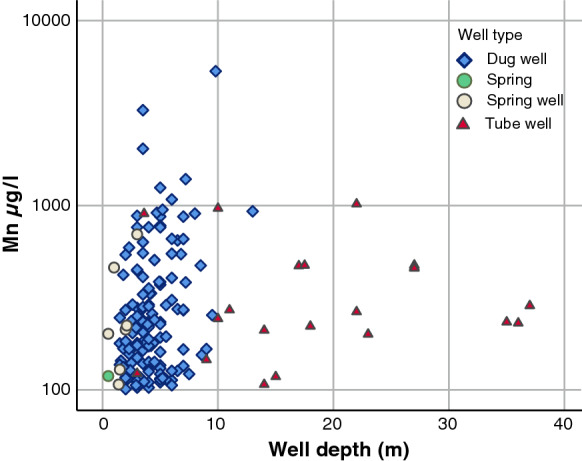
Manganese concentrations in single samples of water from Quaternary deposits wells (Mn > 100 µg/l) according to depth

The number of dug wells was multifold compared to the number of other wells. Most water samples from dug wells were derived from shallow wells below 10 m in depth (Fig. 9). Tube wells were deeper than other Quaternary deposit wells. However, the depth of wells had no clear association with the Mn concentration in single samples of well water from Quaternary deposits (*p* = 0.065).

Fe, pH, and O_2_ levels varied significantly between Mn concentration classes. Median Fe concentrations increased concomitantly with Mn concentrations, being 0.52 mg/l in the highest class of Mn, > 400 µg/l (*p* = 0.00) (Table 5), which is above the quality recommendation for household water Fe of 200 µg/l (0.20 mg/l) in Finland (STM 1352/2015). pH values increased slightly from 6.3, being the highest (6.6) in the two highest Mn classes. Median concentrations of dissolved oxygen decreased from 73.3% to 27.2% as Mn concentrations increased (*p* = 0.00) (Table 5).

**Table 5:** Median, mean, standard deviation (SD), range, and number of samples (*n*) for Fe, pH, and O_2_ in single samples of Quaternary deposit well water in Finland in 1992–2013 according to categories of Mn concentrations

Mn	Fe mg/l^a^	pH (field)^b^	O_2_% (field)^a^
< 50 µg/l			
Median	0.03	6.30	73.3
Mean	0.09	6.38	74.4
SD	0.29	0.61	30.07
Range	< 0.03–9.33	3.40–9.0	5.40–343
* n*	2402	2150	1885
51–100 µg/l			
Median	0.12	6.30	48.50
Mean	0.36	6.35	50.8
SD	0.55	0.63	26.4
Range	< 0.03–3.57	4.8–8.1	2.5–131
* n*	162	141	135
101–400 µg/l			
Median	0.23	6.60	37.7
Mean	0.75	6.59	43.4
SD	1.44	0.64	26.3
Range	< 0.03–10.7	5.1–9.0	0–131
* n*	226	200	194
> 400 µg/l			
Median	0.52	6.60	27.2
Mean	7.14	6.61	32.5
SD	22.0	0.57	21.3
Range	< 0.03–133	5.1–7.8	2.5–85.9
* n*	96	82	78

^a^Kruskal–Wallis test, *p* = 0.00

^b^ANOVA, *p* = 0.00

On average, the highest Mn and Fe concentrations and pH levels and the lowest oxygen concentrations were found in tube wells covered by clay. In Finland, especially in the vicinity of the coast, esker areas are covered with clay layers. Deep wells and tubes were dug through these clay layers to reach the sand layer. The oxygen levels are often low and Fe and Mn concentrations are high in these types of wells (Lahermo et al. 2002). Dissolved oxygen levels decreased as a function of increasing Mn concentrations. When the oxygen level in water decreases, several elements of geological origin, including Mn, dissolve in the well water due to the long water retention time. Unexpectedly, pH was slightly increased concomitantly with Mn in wells dug in Quaternary deposits. The depth of wells in Quaternary deposits had no significant association with Mn concentrations.

Mn concentrations were low in spring waters. Spring water, especially in small groundwater catchment areas, resembles rainwater and is therefore low in Mn. The longer retention time of groundwater in Quaternary deposits probably causes the release of more elements into the water. Mn dissolution in water was on average higher in wells under anaerobic conditions in clay areas than under aerobic conditions in wells dug in sand deposits. The long retention time of groundwater under a clay bed together with the reducing conditions may increase Mn dissolution (Lahermo et al. 1990). In general, the highest Mn concentrations in groundwater are found in the western and southwestern coastal areas of Finland, where confining aquifer conditions and dissolved organic material in the water cause oxygen depletion. Consequently, the aquifer type, for instance, the occurrence of peat and fine-grained sediment, the conditions in the well, and pollution are pronounced contributors to the Mn (and Fe) concentrations (Lahermo et al. 1990). In this study, in single water samples from Quaternary deposit wells, high Mn concentrations were found in coastal areas in the southwestern part of Finland, but also in some wells in northern parts of the country (Fig. 8).

### Quaternary deposits: monitoring sites

**Fig. 10:**
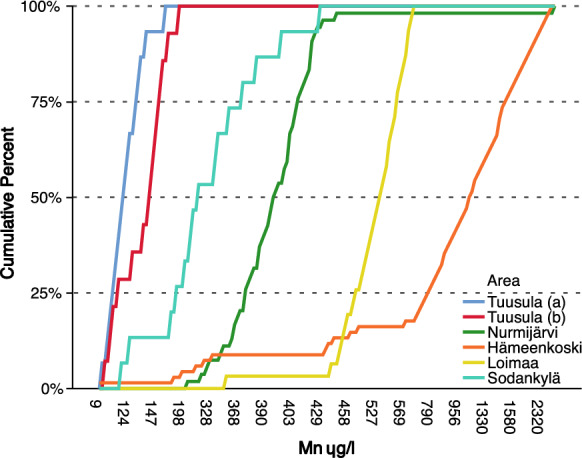
The cumulative percent distribution of manganese concentrations in water from six Quaternary deposit wells where the Mn concentration exceeded 100 µg/l. The Sodankylä monitoring site is located in northern Finland and the others in the southern part of the country

A total of 4227 analyses from 119 Quaternary deposit wells were included in this study. Only 5.8% of the water samples exceeded the national quality recommendation of 50 µg/l for Mn. At some monitoring sites, Mn concentrations in well water remained stable over the years. However, Mn concentrations in well waters from the monitoring sites in the municipalities of Loimaa and Hämeenkoski varied annually, seen as a low gradient curve (Fig. 10). High Mn concentrations, exceeding 100 µg/l, were found in water from six Quaternary deposit wells at monitoring sites: five in southern Finland and one in the northern part of the country.

**Fig. 11:**
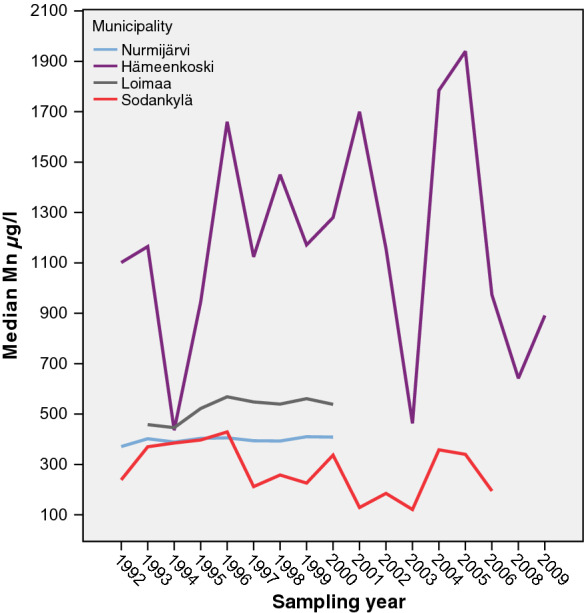
Annual median Mn concentrations in water from Quaternary deposit wells (Mn > 100 µg/l) at monitoring sites in 1992–2009

**Fig. 12:**
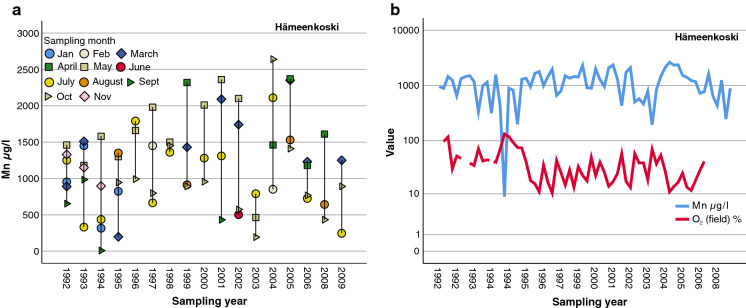
**a** Annual and monthly variation in Mn concentrations in dug well water from the Hämeenkoski monitoring site (min = 8.77 µg/l, max = 2640 µg/l) **b** Annual variation in Mn and O_2_ concentrations in dug well water from the Hämeenkoski monitoring site in 1992–2008

Large annual variation in Mn concentrations in groundwater between the years 1992 and 2009 was found in Hämeenkoski in southern Finland (Figs. 11 and 12a). Water samples were taken from a 3.3-m-deep dug well with concrete rings (Backman 2004). In general, the minimum Mn values varied annually between 193–1410 µg/l in this well water, except in September 1994, when for an unknown reason the minimum Mn concentration was only 8.77 µg/l. The maximum concentration varied from 790 µg/l in October 2003 to 2640 µg/l in October 2004 (Fig. 12). The recharge area of this dug well was heavily burdened. The area was characterized by intensive agricultural activity, including a large cowshed, cattle pastures, a greenhouse, and several residential buildings (Backman 2004), and it remains uncertain whether that contributed to Mn levels. The monthly variability in Mn concentrations in water was large. The highest concentrations were normally found between March and May. In Nurmijärvi in southern Finland, the Mn concentration in spring water during 1992–2000 varied between 215 µg/ (Feb 2000) and 438 µg/l (May 1996). The highest concentration, 3750 µg/l, was measured in July 2000.

Mn concentrations were probably affected by the O_2_ levels in the well water (Fig. 12b). When the oxygen level decreased, more Mn dissolved in the well water.

**Fig. 13:**
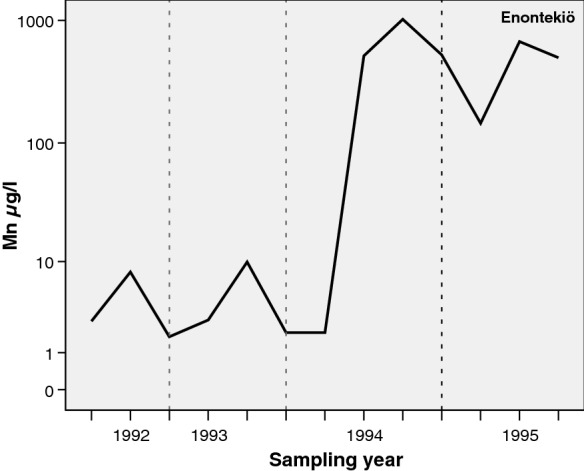
Annual variation in the Mn concentration in dug well water from a till area at the monitoring site in Enontekiö in 1991–1995

High annual variation in the Mn concentration was found in the dug well at the Enontekiö monitoring site, a municipality in Lapland in the northern part of Finland (Fig. 13). The well was deepened by excavation in 1994, and after this, the quality of the well water substantially changed. The pH decreased, being 5.0 at the lowest, but the Mn and Fe concentrations increased, being 1020 µg/l and 1.04 mg/l, respectively. The well probably reached a layer with reducing conditions, or its excavation reached a Fe–Mn precipitation layer. A few years later, after excavating the well, the Fe concentration decreased, while the Mn concentration remained at a high level. Erickson et al. (2019) observed that well construction disturbs the aquifer and the geochemical balance of the groundwater and that the resulting geochemical disturbance affects the arsenic concentrations measured in the wells in northern–central parts of the USA. A similar phenomenon was recorded for Mn concentrations in the dug well in Enontekiö, northern Finland, after the well was excavated to make it deeper.

Mn dissolution in water was on average higher in tube wells located in sandy aquifers covered with clay than under aerobic conditions in wells in sand areas, as shown in Table 6. The retention time of groundwater under a clay bed is long, and conditions are reducing.

**Table 6:** Median, mean, standard deviation, maximum, and 2% and 98% percentiles for Mn concentrations in well waters from different soils layers at monitoring sites in 1992–2009

Quaternary deposit wells			
Mn µg/l	Tube wells covered by sand and clay	Wells in a till area	Wells in a sand area
2%	0.03	0.05	0.04
Median	1.85	0.86	0.90
Mean	49.1	34.6	14.6
SD	135	180	99.1
98%	561	714	358
Maximum	583	1660	3760
*N*	437	1135	2248

In wells with under 100 µg/l Mn, the variability was smaller. The largest variation in this group was in a dug well in Inari, in which the minimum was 0.97 µg/l (Aug 2003) and the maximum 97.40 µg/l (March 1994).

### Total manganese and soluble manganese

**Fig. 14:**
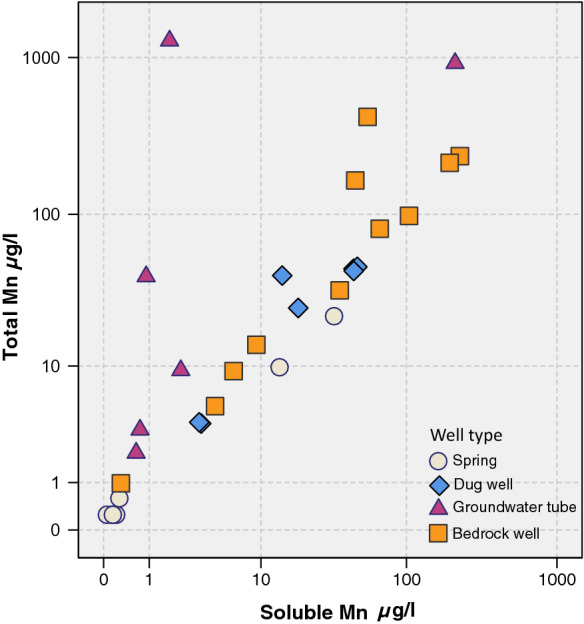
Total Mn (µg/l) concentrations in relation to soluble Mn (µg/l) in groundwater according to the water source in 2005–2013 (*n* = 31)

The soluble form of Mn was typically analyzed in groundwater studies conducted by GTK. Experimental paired analyses for total Mn and soluble Mn were conducted separately from 31 samples, comprising 11 samples from drilled wells and 20 samples from Quaternary deposit wells (Fig. 14). The findings from these analyses indicated that in most samples, the total Mn concentration was higher than the soluble Mn concentration, suggesting that Mn in water was also partly in a particle-bound form. Mn only existed in a purely soluble form in a few wells. The median proportion of soluble Mn was 81.8% in drilled well waters and 56.0% in Quaternary deposit well waters, but the variation was high (Table 7). For wells dug in Quaternary deposits, the water samples were mostly taken directly from the water source, not from a tap. Thus, Mn was already particle bound in the well. If a water sample is taken from a tap, Mn may additionally bind to particles in the water piping system. The notable difference between soluble and total Mn concentrations suggests that to obtain the overall Mn status of the well water, measurement of both soluble Mn and total Mn is preferable.

**Table 7:** Soluble and total Mn concentrations, and median, minimum and maximum content in single samples of water from Quaternary deposit wells and bedrock wells in 2005–2013

	Mn µg/l, soluble	Mn µg/l, total	Mn soluble /Mn, total %
Drilled wells			
1	45.3	165	27.5
2	9.3	14.0	66.1
3	54.8	420	13.0
4	227	236	96.2
5	35.5	32.3	> 100^a^
6	0.30	0.98	30.6
7	4.46	5.1	87.1
8	104	97.9	> 100^a^
9	66.2	80.9	81.8
10	6.3	9.2	67.9
11	194	214	90.7
Total *N*	11	11	
Median	45.3	80.9	81.8
Minimum	0.30	0.98	13.0
Maximum	227	420	> 100^a^
Quaternary deposit wells			
1	1.7	1280	0.1
2	0.74	3.3	22.2
3	14.2	40.3	35.2
4	0.27	0.59	45.8
5	0.91	39.8	2.3
6	0.21	0.25	84.0
7	2.24	9.3	24.0
8	18.4	24.7	74.5
9	0.15	0.25	60.0
10	3.3	3.8	86.9
11	0.64	2.1	30.6
12	3.4	3.7	91.4
13	46.8	45.9	> 100^a^
14	13.6	9.8	> 100^a^
15	44.1	44.6	98.9
16	44.2	43.2	> 100^a^
17	211	921	22.9
18	32.5	21.8	> 100^a^
19	0.13	0.25	52.0
20	0.05	0.25	20.0
Total *N*	20	20	
Median	2.8	9.6	56.0
Minimum	0.05	0.25	0.1
Maximum	211	1280	> 100^a^

^a^According to laboratory: > 100% is due to inaccuracy and uncertainty of measurements

The present data confirm previous findings (Lahermo et al. 1990; Karppinen et al. 2012) and indicate that Mn concentrations are higher in water from private bedrock wells than in Quaternary wells, and high concentrations are rather common in Finland. Similar observations for well waters have been made elsewhere. For example, in Scotland, elevated Mn concentrations, > 50 µg/l, were detected in 30% of 475 groundwater sampling sites (Homonick et al. 2010). In 9% of the water samples analyzed in this study, the Mn concentration exceeded 400 µg/l. For comparison, in Bangladesh the median Mn concentration has been as high as 734 µg/l in wells over 50 m deep, and 126 µg/l in shallow wells below 50 m in depth (Rahman et al., 2017).

## Summary and conclusions

The association of geochemical factors with the natural concentrations of Mn in groundwater was investigated. High Mn concentrations were more common in waters in wells drilled in bedrock than well waters in Quaternary deposits. Among single samples, low dissolved oxygen content associated with high Mn concentrations and elevated Mn with elevated Fe concentration. No clear association was observed between the Mn concentration and pH or depth of wells in single water samples from wells in bedrock or Quaternary deposits.

Among monitoring wells in both bedrock and Quaternary deposits, two different types of wells were found in relation to the Mn concentrations in water: Mn concentrations were stable in the majority of wells, but in addition there were also wells with high variability in Mn concentrations, especially in Quaternary deposits. The Mn concentration did not depend on depth of the well. The annual or seasonal variability in Mn concentrations in well water with high Mn concentrations (> 100 µg/l Mn) was large. High Mn concentrations were more often detectable in the water of bedrock wells than in Quaternary deposit wells. Seasonal variation in Mn concentrations was not common in bedrock wells, but was nevertheless detectable in two wells at monitoring sites.

The total Mn concentration was generally higher than the soluble Mn concentration when both were measured, suggesting that Mn was partly bound to particles in the water, and the relationship between the total and soluble concentrations varied. Thus, the analysis of soluble Mn alone may not always reveal the actual Mn status of the well water. Furthermore, single measurements may not reveal the true Mn status of the well water, and repeated measurements of both soluble and total Mn may be preferable.

The number of wells at monitoring sites was relatively small, and they were located in certain small areas of Finland. Therefore, it is not as yet possible to state how common this variation is. The results are tentative, and a larger number of wells need to be examined to confirm the proportion of wells with high variation in the Mn level.
